# Meta-Intelligence: Understanding, Control, and Interactivity between Creative, Analytical, Practical, and Wisdom-Based Approaches in Problem Solving

**DOI:** 10.3390/jintelligence9020019

**Published:** 2021-04-02

**Authors:** Robert J. Sternberg, Vlad Glaveanu, Sareh Karami, James C. Kaufman, Shane N. Phillipson, David D. Preiss

**Affiliations:** 1 Department of Human Development, Cornell University, Ithaca, NY 14853, USA; 2 Department of Psychology and Counselling, Webster University Geneva, 1293 Bellevue, Switzerland; glaveanu@webster.ch; 3 Centre for the Science of Learning and Technology, University of Bergen, 5007 Bergen, Norway; 4 Educational Psychology Faculty, Mississippi State University, Starkville, MS 39762, USA; skarami@colled.msstate.edu; 5 Neag School of Education, University of Connecticut, Storrs, CT 06269, USA; james.kaufman@uconn.edu; 6 Faculty of Education, Peninsula Campus, Monash University, Frankston 3199, Australia; shane.phillipson@monash.edu; 7 Escuela de Psicología, Pontificia Universidad Católica de Chile, Macul, Santiago 7820436, Chile; davidpreiss@uc.cl

**Keywords:** analytical skills and attitudes, constraint, creative skills and attitudes, intellectual skills and attitudes, meta-intelligence, practical skills and attitudes, problem-solving, solutions, systems, WICS, wisdom-based skills and attitudes

## Abstract

A deeper understanding of the processes leading to problem framing and behind finding solutions to problems should help explain variability in the quality of the solutions to those problems. Using Sternberg’s WICS model as the conceptual basis of problem solving, this article discusses the relations between creative, analytical, practical, and wisdom-based approaches as bases for solutions to problems. We use a construct of *meta-intelligence* to encompass understanding, control, and coordination between these constructs. We propose that constraints can act at each of three levels—individual, contextual, and interactive. Individual constraints include the metacomponents (executive processes) that underpin each of the four kinds of solutions. Contextual constraints direct which of the four approaches are preferred under what circumstances. Finally, interactive constraints involve individual and contextual constraints directly impacting each other’s actions. The model of meta-intelligence and its functioning helps to explain the variability in the ways that individuals frame problems and, as a consequence, in the solutions that are found. The model of meta-intelligence also helps explain why some solutions to problems are so much more comprehensive, and often better, than others.

## 1. Introduction

The world is beset with problems unlike those seen in the recent past. On one level, the COVID-19 pandemic has focused attention on world leaders and governments, and their joint responsibility to manage the tensions between economic growth and the health of people. On another level, business owners have needed to rethink their operational plans to ensure that, at least in the short term, they remain financially viable. Finally, individuals seek to maintain balance in their lives to ensure that they remain physically, mentally, and financially healthy. In solving important life problems, people need to think creatively to generate solutions to problems different from those to which they are accustomed; think analytically to understand the problems and evaluate their solutions; think practically to implement problem solutions and convince others of their value; and think wisely to ensure that their ideas benefit others beside themselves and their allies. Although defining and solving problems is a highly individualized cognitive process, the environmental context shapes not only the nature of problems but also how they are solved. For example, the COVID-19 pandemic has set contextual constraints on finding and keeping jobs, on going to school and college, and most generally, on socializing, that did not exist even a year ago.

The purpose of this article is to discuss a concept of meta-intelligence as a way of understanding the relations of control and coordination among creative, analytical, practical, and wisdom-based approaches to problem solving (Sternberg n.d.b). An approach involves a complex of intellectual skills and attitudes (see also Sternberg 2003) applied to one or more problems. Skills refer to how well someone does something. We view abilities as constellations of skills that tend, at the level of individual differences, to be positively associated with each other. Both skills and abilities, are modifiable, to some extent, both intragenerationally (Ericsson and Pool 2017; Sternberg 1999) and intergenerationally (Flynn 1987). Attitudes refer to propensities to use the relevant skills. Someone could be good at something—for example, creative skills—but as a result of attitudes, choose not to use those skills, perhaps to avoid being ostracized for going against a group norm. Or someone could have an attitude to use certain skills but not be particularly proficient in the use of those skills—as when someone wants to be creative but has non-novel, or common ideas.

We use Sternberg’s (2003, 2019a) WICS (wisdom-intelligence-creativity-synthesized) model to consider the interplay between creative, analytical, practical, and wisdom-based intellectual approaches to problem solving, and to suggest how these four approaches interact with each other and with the environmental context to enhance or diminish effective problem solving. In this model, creative skills and attitudes are used to generate novel, meaningful solutions to problems; analytical skills and attitudes to ascertain whether the ideas are good ones; practical skills and attitudes to apply the solutions and persuade others of their value; and wisdom-based skills and attitudes to ensure that the solutions help to achieve a common good, over the long as well as the short term. Problem-solvers choose one or more approaches to problem solving based on their skills and attitudes as these interact with the problem or problems at hand. The complex of processes of choosing one or more approaches, controlling them, and coordinating the various approaches is what we call *meta-intelligence* (a concept introduced in Sternberg n.d.b). The processes are not always applied consciously. Choices may be made, often without one’s knowing why (Wegner 2017; Wilson 2004).

Consider the essential features of the four approaches. In each case, relevant attitudes influence how one uses one’s meta-intelligence to choose to utilize the relevant skills in problem solution:

1. **Creative** skills are used to generate novel, high-quality solutions to problems.
2. **Analytical** skills are used to ascertain whether solutions (one’s own and others’) are logically sound, internally consistent, and conceptually well-founded. They are overlapping with the rational-thinking skills studied by Stanovich (2010); Stanovich et al. (2018) and others.
3. **Practical** skills are used to ensure the solutions are workable, given the constraints of the real world; to implement the solutions; and to persuade others of the viability of the solutions.
4. **Wisdom-based** skills are used to ensure that the solutions help to achieve a common good, by balancing one’s own, others’, and larger interests, over the long as well as the short terms, through the infusion of positive ethical values (Sternberg 1998, 2003, 2019b, 2020a).

In our model creative, analytical, practical, and wisdom-based intellectual approaches can—or, at least, *should*—work together through meta-intelligence. However, as we shall now see, these windows onto thought and problem solving sometimes come up with different courses of action. The approaches actively work, through meta-intelligence, to constrain each other, both positively and negatively.

This view is rather different from that of both traditional views and some modern views of the intellectual skills that comprise approaches to problem solving, as broadly defined. With regard to historical and contemporary psychometric views (see Kaufman et al. 2020), intellectual functioning generally has been defined more narrowly, mostly taking into consideration analytical abilities.

Our view takes a systems approach (Sternberg 2020c), where different systems of cognitive functioning interact with each other to produce adaptive responses to the environment. Sternberg’s (2019a, 2020a) model differs from Gardner’s (2011) systems model in not envisioning separate intelligences or separate “anythings,” but rather, a system that is completely interactive. The executive (and also the metacognitive) processes involved in creative, analytical, practical, and wise thinking are largely the same. What differs is the purposes for which they are employed (Sternberg n.d.b). As they serve different purposes, they are interdependent in promoting and protecting their own distinctive purposes.

## 2. Four Windows into Problem Solving

We begin with a vignette (Sternberg n.d.a) that has been used to illustrate the essential features of Sternberg’s augmented theory of successful intelligence (Sternberg 2020a). Although fictional, the vignette is plausible, given the challenges facing the world in late 2020:

*A new virus originally appeared in a remote part of the world. Spreading quickly, the world is now experiencing a pandemic with consequences affecting both the physical health of people and the world’s economic stability. In response, the Vaccimax Company has been working within a very tight deadline and under the strict regulatory standards in the U.S. to develop and prepare for distribution a vaccine against the virus. The Chief Operating Officer, Cheryl James, has the responsibility to oversee the project. You are her chief advisor. She is gearing up to enter Phase 3 of the project—randomized controlled trials involving humans. However, Cheryl is facing two problems, on which she wants your advice: First, the company does not have sufficient money to conduct the trials. Second, Vaccimax is using outdated technology and it is not clear whether it could create sufficient quantities at the necessary purity in order to complete Phase 3. If Vaccimax does not complete Phase 3, it will lose the considerable sum of money it has already spent in vaccine development and may go bankrupt. The company has approached their bank for extended loans. However, these efforts have proved unsuccessful because their bank has already given Vaccimax preferential interest rates and views the project as too risky. Other banks are out of the question for fear of souring relations with their current bank. On the up-side, Cheryl has preliminary data indicating that, of 200 people who were given the vaccine, only 10 became sick with the virus one month later, suggesting a failure rate of just 5%. A pharmaceutical company in a foreign country operating under a different regulatory regime has offered to market and distribute the vaccine in this country in return for 50/50 share of the profits. If required, the arrangement can remain confidential. Assuming the marketing by the foreign pharmaceutical company is successful, the profits should be sufficient to fund the Phase 3 trials in the U.S. Cheryl has to decide whether to authorize the foreign company to go ahead with this plan. Please advise Cheryl on what to do and why.*

We suggest that the problem could be viewed through four different windows. Each window serves as a unique lens that focuses on some elements of the problem at the expense of other elements, leading to the recognition and definition of a somewhat different problem and, consequently, of different solutions. We identify the four windows as A = analytical, C = creative, P = practical, and W = wisdom-based perspectives on the problem Cheryl faces (Sternberg 2003, 2019a). Meta-intelligence is used to understand, control, and coordinate the use of the windows.

### 2.1. Window A (Analytical Intelligence)

Young people most commonly learn to use Window A in their education, including in business schooling. Entering into an agreement would be folly because the trial is flawed. There is no control group in the available data. One cannot conclude that the vaccine failure rate was 5% because one does not know what number of people would have gotten the disease in an unvaccinated control group. The solution for Vaccimax is to be more carefully analytical in evaluating the data they have so they are not fooled by results lacking a control group.

### 2.2. Window C (Creative Intelligence)

Entering into an agreement would be folly until the company has a better technological procedure for creating vaccine of sufficient quantity and quality. Hampered by its outdated technology, Vaccimax needs a creative breakthrough that would enable Vaccimax to produce more and higher quality vaccine. The creative solution will ensure that, if the vaccine succeeds, Vaccimax can produce the vaccine in sufficient quantities to make money.

### 2.3. Window P (Practical Intelligence)

Entering into the agreement would be folly because there is no guarantee that money will overcome the issues of vaccine quality and quantity. Cheryl knows of other companies that are developing vaccines against the virus and believes that a license for the marketing of their vaccines could be negotiated. Cheryl understands that such an arrangement would limit the immediate investment costs but would also reduce the company’s future profits. Moreover, entering into the agreement would be folly because, practically speaking, it is probably illegal, raising the specter of costly civil litigation and possibly criminal charges. Although confidentiality is offered, it is likely that leaks would occur and would be viewed negatively by the public as a run around U.S. regulators and thus could expose the company to huge penalties. Further, Cheryl needs to have a serious conversation with the CEO about approaching other lenders, not necessarily banks. A venture-capital company might be willing to support Phase 3 in return for a stake in the company. Given the huge potential profits of a successful vaccine, any number of venture capitalists might be interested. A better alternative might be to outsource testing and production of Vaccimax, although doing so would mean the company’s taking a financial hit.

### 2.4. Window W (Wisdom)

In the present day, Window W is seriously under-utilized. First, entering into the agreement would be folly because it breaches Cheryl’s moral values and those of the company. For Cheryl, the agreement circumvents regulatory standards that are designed to ensure patient safety. Second, Vaccimax would be risking the health and wellbeing of all those in the foreign country who take the vaccine. Third, the company is risking their ethical heritage and the values that inspired their founders to create a company serving the common good. For good measure, the company needs to check whether the protocols used in the collection of the preliminary data on 200 individuals produced any long-term hidden side effects.

Each window on Cheryl’s thinking illustrates different aspects of the problem and their potential solution. In this case, Cheryl’s viewing the problem through different windows leads to the same conclusion—not to pursue the deal—but for entirely different reasons. Had the details of the case been different, Cheryl might have come to a different conclusion as a result of peering through each window. For example, the trial with 200 individuals might have been conducted in a more rigorous way with a control group, allowing a positive decision through Window A, or the technology for producing larger quantities of a purer vaccine might have been better developed, allowing a positive decision through Window C. In such a case, the same windows could have illustrated conflicting recommendations for a course of action.

Sometimes, people, and especially leaders, view Window W as taking priority over all over windows. In 1982, some bottles of Tylenol pain-reliever were intentionally spiked with poison. The CEO of Johnson & Johnson at the time, James E. Burke, decided to recall *all* bottles of Tylenol, even though the event appeared to be limited to a local geographic area. There were creatively, analytically, and practically viable marketing campaigns that could have made clear that the poisoning was in a limited geographic area. Burke recalled all the Tylenol bottles anyway, as a message that the company put its ethical responsibilities above its profit-making responsibilities. In the long run, the move probably helped Johnson & Johnson by burnishing the reputation of the brand.

In the context of a pandemic, wise solutions seem to be especially adaptive. Sharing of scientific information, resources for treatment, and vaccines globally is not only wise, but also is adaptive because, in the context of an infectious and highly transmissible disease circulating across a highly interconnected and interdependent global economy and world, no one anywhere will properly feel or be safe. Moreover, no one will fully recover their personal wellbeing until the disease is contained across all of the five continents, a containment which will require international collaboration. Vaccine nationalism will take the world, and has taken the world, to a worst-case scenario and, ironically, seems most to harm those who intend to shield themselves from others, as in the United States of 2020.

The choice of windows and the efficacy of their utilization depend on many different factors. Abilities and preferences will certainly play a role, but these are in turn affected by environmental variables, such as opportunities to acquire knowledge, the kinds of knowledge one is likely to acquire in given environments, innumerable sociocultural variables that would be difficult to quantify, and, of course, the genetic predispositions with which people are born. In our view, abilities and interests are largely acquired and shaped through experience to develop into competencies and expertise (Sternberg 1999).

It may seem, at first thought, that the four windows and their interrelations present so much complication that they never plausibly could be used by one person at one time for one problem. Yet, many of us use all four windows in daily life, even in solving mundane problems. For example, one of us with a pre-adolescent child discovered that the child had sneaked her iPad into her bed. First, the parent tried to analyze what the child was trying to do. As it turned out, she was checking and answering social-media posts. Second, the parent needed a creative idea to make sure it would not happen again. The child was told that she certainly did not need the iPad both day and night, so if she ever brought it to bed again at night, she would lose the use of it the next day. Third, the solution had to be practical. It seemed to be, because the child used the iPad so heavily during the day that she would not want to be deprived of the opportunity to use it. Finally, the decision had to be wise. The parent hoped it would be, because it would benefit not only that child, but the two siblings who were told that the same rule would apply to them. This way, the children would not be deprived of sleep because they were secretly on their iPads late into the night.

The example is quite trivial, but that is exactly the point. In our daily lives, we use the four windows even to solve the mundane problems that face us on a daily basis. Of course, the four windows are often used collaboratively, as in the above case, in which the coauthor and his wife collaboratively arrived at the decision to implement the plan as described.

Meta-intelligence also is used collaboratively. Examples of how meta-intelligence can be used collaboratively stem directly from research on group intelligence (Williams and Sternberg 1988), collective intelligence (Malone and Woolley 2020), group creativity (Paulus and Nijstad 2003; Sawyer 2003), and the wisdom of crowds (Surowiecki 2005).

The operations of meta-intelligence are through metacomponents that extend not just to intelligence (the use for which they originally were proposed—Sternberg 1985a), but to creativity and wisdom as well. How, exactly, do they operate—for example, in any particular order, or with any particular weighting? Meta-intelligence is heterarchical, not hierarchical as are mental processes in the process model of Bloom (Anderson and Krathwohl 2000) or the mental abilities in the structural model of Carroll (1993) and other hierarchical theorists of intelligence. In the current model, processing is iterative and somewhat unpredictable. For example, a creative idea may give rise to the need to analyze whether the idea is truly tenable, which in turn may give rise to questions of whether it is practical and wise, which in turn may lead one to revise the idea and ask whether the revision is tenable, and so forth. There is no fixed order, and no fixed weighting. Rather, these aspects of processing must be determined as a function of the problem and the situation in which it is solved. The meta-intelligence is in the act of sequencing and, often, re-sequencing, so as to optimize the quality of the outcome.

There will be individual differences in meta-intelligence, of course, and in patterns of usage of the windows. These individual differences will derive from the interactions of prior knowledge and abilities with problems and with situations. Environmental experience will be important. For example, if scholastic reward systems heavily favor analytical problems and analytical solutions to problems, and if individuals have done well under such systems, they will be primed through past reinforcements to approach problems in analytical ways. If individuals are creatively disposed and have been adequately rewarded for creative solutions to problems allowing creativity, they may be more inclined toward creative solutions. If, instead, they have been nonreinforced or punished for creative solutions, they may be less likely to try them out, especially if they were not originally of a disposition to gravitate toward creative solutions.

## 3. Processes for Solving Problems

Whether Cheryl uses her meta-intelligence to solve the problem through the analytical, creative, practical, or wisdom-based window, or some coordinated combination of some or all of these, the processes she needs to solve the problem are roughly the same. The processes will give different solutions depending on the window she uses, but they are the same, nevertheless.

Sternberg’s (2019a, 2020a) augmented theory of successful intelligence considers each of the four separate elements as being served by the same executive processes, or metacomponents (Sternberg 1985a). These seven metacomponents include (1) recognizing the existence of a problem, (2) defining the problem, (3) allocating resources to the solution of the problem, (4) mentally representing the problem, (5) formulating a strategy to solve the problem, (6) monitoring the success of the strategy while it is being used, and (7) evaluating the strategy after it has been employed. Problem recognition and definition are elements that sometimes, in the past, have been referred to as “problem finding” (Arlin 1975; Getzels 1979) or “problem construction” (Reiter-Palmon and Robinson 2009).

The first five metacomponents occur before problem solution, the sixth during problem solution, and the seventh after problem solving is completed. Similar processes have been suggested by others (e.g., Bransford and Stein 1993; Mumford et al. 1991).

In order to optimally solve the problem presented to Cheryl, a high level of meta-intelligence demands that all four approaches have to be used collaboratively, with the recognition that they will sometimes come into conflict. Within an organization, people can capitalize on their own strengths and compensate for their weaknesses by working on interdisciplinary teams. Although it is expected that wisdom-based skills and attitudes are present in all the members of an organization, companies working on critical issues, such as developing a vaccine, undoubtedly benefit from having specialized units in charge of the ethical oversight and the social and environmental sustainability of their operation. The existence of these units, many times, is a consequence of a deliberate decision or of regulation and external pressures by the government, the public, or stakeholders.

In the case of Cheryl, the different windows lead to different strategies. The strategies might be used in conjunction, or some might be preferred to others, depending on the end goals or circumstances. Regardless, anyone who consistently looks only through a single window will be a poor problem-solver. Even if there are some problems that may be solved in a limited way with just one window, most problem-solving processes would benefit from multiple views (or all of them). Yet, schooling very much emphasizes the analytical window, perhaps because it is most relevant to the kinds of not very consequential and often artificial problems presented on standardized tests, which are the gatekeepers for university entrance in some countries. Additionally, this emphasis on analytical skills was favored in the education of those in charge of educating new generations. They, like other people, tend to resort to the lessons of their own experience in searching for models for their own thought patterns and behavior and for the thought patterns and behavior they teach to others.

A given window does not lead to a unique solution or even problem-solving process. Rather, a window is a perspective on how a problem should be defined. Within that definition, many different solutions could be reached. Windows interact with problems, situations, and aspects of the person—pattern of abilities, personality, and motivation—to produce solutions that represent the complexity of the forces inside and outside—context-based—of the person to do what seems to need to be done to solve a problem. Different theories specify different ways in which these interactions might take place (Danner et al. 2011; Tett and Burnett 2003; Ziegler et al. 2018).

In particular, Cheryl needs to utilize the seven executive processes or metacomponents (Sternberg 2019a), regardless of which window or windows she uses in problem solving:

### 3.1. Recognition of the Existence of a Problem

She has to recognize that something is not the way it should be. For example, first, from the viewpoint of an analytical window, the company does not have sufficient money to conduct the trials. Second, from the viewpoint of a creative window, Vaccimax is using outdated technology and it is not clear whether it could create sufficient quantities at the necessary purity in order complete Phase 3. Clearly, something is wrong.

### 3.2. Definition of the Problem

How Cheryl defines the problem depends on the window through which she sees it. Through Window C, she sees a new way to create new technology that will enable a high-quality vaccine. Through Window A, she sees folly because the trial is analytically flawed. It needs to be fixed. Through Window P, she sees problems of financing the vaccine research. Where exactly will the money come from? Through Window W, she sees potential ethical problems and the potential failure of the company to act in a way that helps to achieve a common good. In sum, her definition of the problem depends upon the window through which she views the problem.

### 3.3. Allocating Resources to the Problem

Cheryl has decided to hire you to help her solve the problem. She is hoping that you, serving as a resource, will help her arrive at an optimal solution. She is paying you a consulting fee and has indicated she is willing to make company resources available to you to help you make recommendations. The window(s) through which Cheryl views the problem will determine what other kinds of resources, and how much of them, she is willing to invest in the problem. For example, the analytical window leads to a potentially substantial financial investment, the wisdom-based one to an investment of resources to ensure that one’s strategy for solving the problem of vaccine production is ethical. In the wisdom window, short-term financial gain may be sacrificed to the hope of a longer term one. This is no small matter, as COVID-19 vaccines that have not met even the most minimal standards of normal scientific evaluation (Sutton 2020) are right now being placed by various countries on the market.

### 3.4. Representing the Problem

How Cheryl represents the problem will depend on which window or windows she looks into to solve the problem. She may have a simple representation, for example, solely in terms of one window, such as seeing the problem only as one of a flawed trial (Window A) or as of finances (Window P). Or she may look through multiple windows, thereby representing the problem in a more complex way, as having numerous constituent elements, all of which need to be addressed.

### 3.5. Formulating a Strategy to Solve the Problem, Monitoring Problem Solving, Evaluating Problem Solving

Cheryl’s strategy for solving the problem will depend upon the window or windows through which she views the problem. If she chooses Window A, she can plan for a better and unflawed trial. Through Window C, she envisions a plan to create a better technology for producing the vaccine. Through Window P, she sees the need for better financial backing before proceeding. Additionally, through Window W, she asks whether her drive for the vaccine represents only profits for Vaccimax or also a desire to do something good for the world.

Whether or not the solution is viewed as a success will depend in part on the window(s) through which one is peering. Looking through Window W, for example, Cheryl may recognize that the company has lost money in the short term but has avoided potentially disastrous long-term fiascoes. Each window puts her on a different strategic path, or by using her meta-intelligence to view and understand the problem broadly through several windows, she may try out multiple strategic paths.

The main points to be learned from the example of Cheryl are that:

1. **The four perspectives can lead to different viewpoints on, and definitions of the problem.** How the problem is defined depends on the window through which one’s meta-intelligence leads one to view it. One window (A) leads primarily to an analysis of the testing conditions; a second window (C) encourages one to think about more creative technologies; a third window (P) leads one to worry about falling afoul of the law; and a fourth window (W) leads one to ask whether what one is doing helps to achieve a common good.
2. **The perspectives are complementary and synergistic.** Therefore, a high level of meta-intelligence would demand that they ideally should all be considered together. Ideally, a full analysis of the problem would involve all four perspectives viewed in interaction with each other.
3. **Any smaller number of perspectives would define the problem incompletely.** Although any given problem can be solved using only one perspective, problems benefit from taking into account multiple perspectives, such as those of creative, analytical, practical, and wise thinking. This also means that, from the standpoint of meta-intelligence, one has to define a given problem in a way that considers as many windows as possible, because that multi-perspective approach provides a more adaptive solution than if only one is considered.
4. **Taking into account more perspectives may lead to different conclusions, depending on the facts at hand**. This is all the more reason to use all four perspectives, wherever possible, rather than just one or perhaps two. A complete model of problem solving needs to take into account the different conclusions that each perspective yields.
5. **Any one person might tend to focus on one perspective or possibly two at the expense of the others.** People see problems through different windows, although some windows may be more socio-culturally approved or encouraged than others. Our enculturation and socialization may lead our meta-intelligence to emphasize some windows at the expense of others. For example, typical industrial and post-industrial Western instruction and assessment emphasize memory and analytical thinking, so that those individuals who have passed through such schooling may be more likely to use Window A at the expense of the other windows, whose use is much less rewarded. However, just using window A leads to an incomplete consideration of options for problem solving. Or different persons within the same organization or even culture may tend to see a given problem just from their own preferred perspective (window) and as a result of a particular role they play in an organization or society. For example, the windows used by the scientists working at Vaccimax are likely to be different from those used by the accountants. Therefore, creating a common broad ground among all the members of an organization is desirable, whereby problem solvers try to look through all four of the windows, where relevant. It is also why having a diverse workforce and groups that represent different departments and backgrounds in the company is often associated with more innovation and higher productivity (e.g., Yap et al. 2005).

Table 1 gives a further example of a problem that can be solved through the four windows discussed in this article, using the metacomponents described above. The example problem here is being unhappy in one’s work environment.

## 4. Constraints in Problem Solving

How, exactly, would the processes work together in specific contexts? We argue that a proper understanding of their dynamics depends on analyzing the kinds of constraints placed on each process or perspective and on their interaction. These constraints are demands that modulate the expression of each process and come from: (a) the internal relations of the four perspectives (or windows); (b) their external, cultural expression; and (c) their inter-relations in concrete situations. In particular, we identify *individual, contextual,* and *interactive* constraints. The constraints represent a process of internalization, whereby people observe the demands of the environment and then adjust who they are and what they do in response to these demands (Vygotsky 1978; Wertsch 1988). Put another way, people are inseparable from the cultural contexts in which they live.

Individual constraints refer to how the four approaches identified above generally place demands on each other. Each perspective is already shaped by a set of contextual constraints, and especially cultural ones (Cole 1996; Sternberg 2004). Finally, individual and contextual constraints interact with each other in ways that allow perspectives to cooperate or to be in conflict. These are interactive constraints that play an essential part in analyzing all agentic human action and interaction.

### 4.1. Individual Constraints

As one looks at problems through different windows, one set of processes serves as a group of enhancers and is foregrounded in the solution of a problem; another set of processes serves as brakes or diminishers and is backgrounded in the solution. To understand this, we need to start from a general analysis of each process or perspective—without bringing in context for the moment—and how it might impact the expression of other processes.

On this view, all four of these constructs virtually always work, or should work, together. In each case, a certain focus comes to the fore: (1) for creativity, generating something new and meaningful; (2) for analytical intelligence, achieving a conceptually sound understanding and evaluation; (3) for practical intelligence, implementing or having something useful and persuading others of its usefulness; and (4) for wisdom, achieving a common good through the balancing of one’s own, others’, and larger interests over the long as well as the short term. However, depending on which set of processes is involved (creativity, analytical intelligence, practical intelligence, wisdom), some concepts and concerns will be foregrounded and others backgrounded. This dynamic will become obvious when we discuss interactive constraints.

Therefore, each one of these approaches is limited by the others.

Use of an *analytical (academic) approach* should be (but is not, at least as measured on standardized tests) constrained by:

(1) creative skills and attitudes: Is the analysis at all new, or does it essentially just repeat what others have said?
(2) practical skills and attitudes: Does the analysis have any practical implications for anyone--is it even usable in the real world?
(3) wisdom-based skills and attitudes: Is the analysis wise or does it lead to destructive ends, such as the analyses of brilliant psychologists that led to scientific racism and eugenics?

Use of a *creative approach* seeks to find solutions that are novel and meaningful. However, this approach should be constrained by:

(1) analytical skills and attitudes: Is the novel idea a logical, analytically sound, internally coherent, high-quality one?
(2) practical skills and attitudes: Is the novel idea a useful, practical, meaningful, executable one?
(3) wisdom-based skills and attitudes: Is the novel idea representative of light creativity--seeking a common good as ends—or dark creativity—seeking destructive ends?

Use of a *practical* (common sense) *approach* seeks to find solutions that are pragmatic and persuasive. However, use of this approach should be constrained by:

(1) creative skills and attitudes: Is the idea novel or just a rehash of what others have done before?
(2) analytical skills and attitudes: Is the idea conceptually sound and rigorous?
(3) wisdom-based skills and attitudes: Is the idea you are selling going to help anyone besides you, or at least, not hurt anyone else?

Use of a *wisdom-based approach* seeks to achieve a common good over the long as well as the short term. However, use of this approach should be constrained by:

(1) creative skills and attitudes: Is the idea new?
(2) analytical skills and attitudes: Is the idea sound and good?
(3) practical skills and attitudes: Is the idea viable in practice as well as in theory, or is it just wishful thinking?

In the meantime, we need to acknowledge as well that these approaches are always expressed in specific contexts. These contexts, and culture in particular, themselves constrain the manifestation of each process. In other words, what constitutes a creative approach in one geographical space at a certain moment in time might be considered to be lacking in creativity at another time. What is wise for some might seem foolish to others. The interplay between approaches is historically determined by what sorts of limitations are more relevant than others as modulated by context.

### 4.2. Contextual Constraints

Just as there are individual constraints, so are there contextual ones. These constraints take the form of cultural and societal expectations that push certain ideas into the foreground while pulling other ideas into the background. For example, ideas about how to achieve racial equality and equal opportunity for all are foregrounded in the present day. Ideas about eugenics once tended to be viewed as progressive and modern, whereas today they tend to be viewed as reactionary and antiquated. These ideas are viewed as antiquated, at best. Ideas about how better to segregate different races were once viewed—by some Whites, at least—as a way to ensure the forward-looking American dream for new (White) homeowners. Today they are viewed, at least by many, as segregationist throwbacks designed to unfairly hold groups of people behind (Krugman 2020; Rothstein 2017).

These examples show that what is viewed as analytically, creatively, practically, or wisely adaptive varies across both time and place (Sternberg 2021). This is what we call here *content* (what) constraints, or those normative boundaries concerning what passes for creative, analytical, practical and wise behavior in a community, at a given time. As Greenfield (2020) has shown, even what is considered “intelligence” changes over time, and what is intelligent also varies across place (Berry 1974; Cole et al. 1971; Serpell 1974; Sternberg 2004; Preiss and Sternberg 2005). Similarly, what is viewed as creative varies across contexts (Glaveanu 2010; Glaveanu et al. 2019). And similarly, different groups have different conceptions of wisdom and what is wise (Ferrari and Alhosseini 2019; Sternberg 1985b; Yang and Intezari 2019).

To elaborate on the topic of creativity, there will generally be a big difference between the aims of those who wish to act creatively depending on whether they belong to a Western or Eastern cultural tradition (Lubart 1990; Niu and Sternberg 2006). Keeping in mind the danger of overgeneralization, it is likely that creators in the West view creativity as a revolutionary act. It is an expression of individuality that breaks with the old and with tradition. It even can be hindered by too much knowledge (Frensch and Sternberg 1989). In contrast, creators in the East are more influenced by cultural values (Phillipson 2013) that emphasize gradual change, the importance of maintaining these values, the need for authenticity, and need for a mastery of the domain (Kharkhurin 2014; Niu 2012). Of course, we need to consider nuanced differences: Within the West, for example, there are relevant internal differences: To illustrate, conceptions of creativity in Latin America and the Caribbean, both of which are located in the Western hemisphere, are rooted in the hybrid nature of the region’s culture in contrast with the culturally universal conceptions of creativity that are dominant in the Western Northern Hemisphere (North America and Europe; Preiss and Strasser 2006). On the other side of the world, Hong Kong borrows from Western perspectives of creativity but is heavily influenced by belief systems based on Confucianism (Tam and Phillipson n.d.).

The differences above in *what* constitutes creativity (content constraints)—or, for that matter, what it means to be analytical, practical, and wise—impact also *how* people act creatively, analytically, practically, and wisely (process constraints). These process constraints imposed by a cultural context refer to the way in which things are done. If we return to the example of creativity, two Western-based creators who are animated by the goal to generate something highly original can still go about this task in different ways. For instance, some might proceed in an empirical manner, collecting data to test a hypothesis, and others in a more conceptual one, providing the theoretical basis for empirical work (Galenson 2011). Or, depending on the specific domain in which creativity is expressed, creative people will use different tools, knowledge bases, and strategies to reach new and valuable outcomes (Glaveanu et al. 2013; Kaufman and Baer 2004).

Finally, there will be specific times and places that call for creative actions and others that require more conventional responses. These *setting*-driven constraints concern the *when* and *where* of creative action and have been studied so far mostly in terms of creative metacognition, or the knowledge of when (and how) to create and how to best utilize personal strengths and navigate cultural constraints (Kaufman and Beghetto 2013). Metacognition, in addition to helping someone determine the appropriate moment to be creative, can also, depending on the individual, either constrain creativity (Preiss et al. 2016) or enable it (Preiss et al. 2019). The same contextual constraints certainly apply to analytical and practical intelligence and to wisdom. Practical common sense and wisdom in particular depend on adapting to one’s context, both individual and cultural.

The effects of cultural constraints on the “what” (content), “how” (process), and “when/where” (setting) of the processes discussed in this article easily can be seen in the real world. They lead to similarities but also to highly divergent goals adopted by individuals and communities in how they approach one and the same problem. For instance, many countries, including but certainly not limited to the United States, have put economic development at the forefront of their consideration of how to handle the pandemic, seeing it as the practical, wise, and even creative thing to do. Subsequent results have shown that only dealing appropriately with health considerations can protect the economy and the markets from much of the pandemic toll. The comparison between Sweden and their other Nordic neighbors has been commonly mentioned as an illustration that using herd immunity strategies to protect the economy does not produce better economic results than smart public health choices focused on time-sensitive quarantines and testing, tracing, and isolating emerging outbreaks.

In the field of energy, a great deal of thinking has been put into how the processes can be used to foster economic development. For example, fracking and horizontal drilling for oil were two practices in the petroleum industry that seemed to some people, both in and out of the industry, as a great boon. At the same time that some countries, such as the United States, encouraged fracking, others, like France and Ireland, banned it because of potential environmental damage. Additionally, both sides might argue they are intelligent and wise to make these choices. The same processes can be applied to a problem, but the outcome can and does depend on cultural and other values, such as the emphasis on economic development versus environmental protection. These considerations will come to shape the content of what is considered right and wise, constrain the means of action, and regulate its timing as well as its sense of urgency. At the end, however, it will be necessary to strike a balance between the different approaches, taking into consideration that the final arrangement must be compatible with long-term human survival (adaptation). Such goals require consideration of the common good not only of our contemporaries but also of our conspecifics not yet born. Wisdom-free, short-term choices will probably drive us to a situation where we would not only risk economic shortfalls over the long run but also face the possible specter of the end of our species’ survival.

### 4.3. Interactive Constraints

Ultimately, both individual and contextual constraints come to bear on the active expression of one or more processes or perspectives from the four outlined at the start of this article. To recap, *individual* constraints are general in nature and concern the intrinsic interplay between creative, analytical, practical, and wisdom-based approaches and how they interact with each other. *Contextual* constraints point to the role of context for both the constitution and expression of what is creative, analytical, practical, and wise. As we have argued, culture in particular sets up a series of demands on what, how, and when/where the four processes are manifested. However, at the end of the day, their expression will always be situation specific or, rather, come out of concrete encounters between the person/group and the situation at hand.

The person’s approaches—through the four windows discussed here—will necessarily influence each other; the social, material, and cultural context will add to these new types of influences. However, it is at the ‘meeting point’ between individual and world that the two main sets of constraints *interact* and, as a result, guide the adoption (or rejection) of specific perspectives. This is especially true for non-academic skills and attitudes, such as those developed by street children to survive every day, or for practical skills and attitudes that are developed at work, both technical and social, and which are key to success in the real world.

To illustrate, the current COVID-19 pandemic requires a strong investment in creativity as regards vaccine production, but while a vaccine is being produced and distributed, we may require applying more standard and traditional public-health measures such as quarantines or face coverings. We also need to persuade people that they are appropriate. Additionally, these measures have to be applied considering the greater common good so they do not produce health costs that are larger than those they look to mitigate, at the same time that we adjust the measures to the vagaries of a highly interconnected and global society. If the money spent on coronavirus research is so great that all other medical research and procedures come to a standstill, the investment in curing the disease ultimately may impede attainment of a common good. Additionally, if people postpone other health-related treatments because of fear of getting infected at medical facilities, this postponement will not help their overall wellbeing either. If quarantines are too long, they can be negatively impactful on other health conditions (including mental health) and produce such a large impact on the availability of jobs that people may be unable or unwilling to adhere to the quarantines. Society has to strike a balance between protecting people from the new virus and from previous established illnesses, as well as between the need for social distancing to avoid transmission and the need to keep a functioning economy.

## 5. Why Meta-Intelligence?

Why introduce a construct of meta-intelligence to understand the coordination of the analytical, creative, practical, and wisdom-based functions?

First, meta-intelligence provides us with a way in which we can understand our own range and functioning of higher mental abilities. (Conventional or general) intelligence primarily learns, analyzes, and evaluates; creativity primarily creates; wisdom deploys creation and analysis for a common good. Intelligence, creativity, and wisdom are not singular or somehow “pure” abilities. They are not like verbal, quantitative, and spatial abilities, for example. Rather, intelligence, creativity, and wisdom—understood, controlled, organized, and deployed by meta-intelligence—serve different purposes. We understand that we can recognize, define, and solve convergent problems (general intelligence); recognize, define, and solve divergent problems (creativity); and put our solutions to the use of seeking a common good (wisdom).

Second, meta-intelligence provides the means by which we decide upon which set of collections of abilities we use when and how. It allows us to use the abilities that fit a given situation.

Third, meta-intelligence coordinates the use of the different collections of abilities. It provides a means by which we can control the deployment of those collections of abilities. A given problem may require intelligence, creativity, and wisdom, such as of a problem of how to allocate scarce resources, or such as of a new vaccine against an illness such as COVID-19 that has become widespread as a pandemic. Meta-intelligence enables us to know what to do when and then do it.

One reasonably might ask whether individuals have stable preferences among windows for solving problems. In other words, might some people, say, tend to gravitate toward an analytically oriented intelligent solution whereas others might work toward a creative solution? In an ideal world, the window or windows one would prefer would be totally problem- and situation- dependent. That is, one would fit the solution strategy to the problem and the situation in which it is presented. This is unlikely to happen for three reasons. First, people have different strengths and may be susceptible to solving problems with the tools that represent their strengths. Second, people have different preferences. They may simply gravitate toward particular windows, whether or not they are adept in their use. Third, and finally, people may filter perceptions of problems so that they construe the problems in certain ways (Sternberg 1997). Metaphorically, a carpenter might see problems as ones requiring a hammer whereas a painter might see problems as requiring a paint brush.

Do we need a new construct, such as of meta-intelligence? Actually, we have always known that people need to decide what kinds of higher order mental resources they need to allocate to a given problem and control that allocation. Meta-intelligence simply names this construct that always was implicitly there.

## 6. Relation to Existing Constructs

A legitimate concern of readers might be the relation to, and possible overlap between existing constructs and our model, and especially the construct of meta-intelligence. Three constructs with which meta-intelligence might be viewed as overlapping are (a) general intelligence *(g*), (b) broader intelligence (c) metacognition, (d) executive processing, and (e) personality.

### 6.1. Overlap with General Intelligence

Is meta-intelligence the same as general intelligence? General intelligence is at the top of many psychometric hierarchies of intelligence, as in Carroll’s (1993) and McGrew’s (2005) models. These are structural models, so they make no clear and systematic claims about information processing. However, all subfactors lower than *g* in the models contribute, at some level, to *g,* which could be seen as the overarching ability factor for all those subfactors. However, in no existing model of human abilities of which we are aware is *g* a super-factor that encompasses creativity and wisdom—that is, in no existing model are creativity and wisdom subsets of *g.* Such a claim would be extraordinary, because by any serious extant models, creativity and wisdom both encompass far more wide-ranging skills than does general intelligence (see Kaufman and Sternberg 2019; Sternberg 2020b; Sternberg and Glück 2019). For example, general intelligence does not include defiance of the crowd, an essential element of creativity (Sternberg 2018), or seeking of a common good, an essential element of wisdom (Sternberg 1998). Nor do tests of general intelligence measure these constructs.

For instance, the Cattell-Horn-Carroll (CHC) model places creativity within the factor of *Glr*, or Long term storage and retrieval (Schneider and McGrew 2018). *Glr* has recently been split into *Gl* (learning efficiency) and *Gr* (retrieval fluency), with creativity (or, rather, divergent thinking) falling under *Gr*—yet when *Glr* is measured on any intelligence test, it is only the *Gl* component; creativity is not included (Kaufman et al. 2011). Thus, meta-intelligence embraces intelligence, creativity, and wisdom in a way that *g* does not and is not alleged to in existing theories.

A related argument would be that aspects of meta-intelligence are renamings, for example, of practical intelligence as a new name for crystallized intelligence (Cattell 1971). This equation of constructs has already been shown not to hold up, as reviewed by Hedlund (2020; see also Sternberg and Hedlund 2002; Sternberg et al. 2001). Practical intelligence, which is based on tacit procedural knowledge, certainly draws on crystallized intelligence as well as on fluid intelligence. However, the correlations are relatively weak across many different testing situations, even if one corrects for attenuation and restriction of range. Or perhaps wisdom is crystallized ability plus a willingness to use this ability. The problem is that people may be knowledgeable but unwise, because wisdom, according to every extant theory of the construct of which we are aware, entails not just crystallized intelligence and “willingness,” but effective deployment of that knowledge; in some theories, that deployment is toward a common good (e.g., Sternberg 1998). People may be knowledgeable but not interested in, or skilled at, deploying their knowledge for a common good. Rather, they may use their crystallized ability, or practical intelligence, just for their own good. The world sees a lot of that.

### 6.2. Overlap with Broader Theories of Intelligence

Is meta-intelligence the same as intelligence, construed broadly? The proposed account here is consistent with broader systems theories of intelligence (Sternberg 2020c), such as Gardner’s (2011) theory and especially Sternberg’s (2020a) augmented theory of successful intelligence. However, Sternberg’s (2003, 2020a) earlier work specified that creative, analytical, and practical intelligence, as well as wisdom, are part of a broader conception of successful intelligence, but it lacked any mechanism for these constructs to interact and work together. That is, they were presented as separate elements—creative intelligence to formulate novel and useful ideas, analytical intelligence to specify whether the ideas were tenable, practical intelligence to implement the ideas and persuade others of them, and wisdom-based skills to ensure a common good. The augmented theory of successful intelligence not only had no mechanism for how the elements worked together, it also dealt only with creative intelligence, not creativity (which includes elements of personality, motivation, and environment as well as of cognition).

### 6.3. Overlap with Metacognition

Is meta-intelligence the same as metacognition? Metacognition is usually defined as comprising one’s understanding of, and control of one’s cognition. Metacognition is important to the effective execution of many, if not most cognitive operations. One needs to put them together. However, metacognition is not the same as what we are calling meta-intelligence, because so much of creativity and wisdom are either affective, attitudinal, or motivational. For example, defying the crowd is an attitude; it is not cognitive. Similarly, intrinsic motivation, a crucial part of creativity in many theories (see, e.g., Hennessey 2019), is not cognitive but rather conative. Similarly, emotional sensitivity and regulation, an element of the MORE model of wisdom (Glück and Bluck 2013), as well as other models, is not a cognitive but an affective attribute. In other words, metacognition simply does not encompass the scope of meta-intelligence as defined here, which manages creativity and wisdom as well as intelligence and its interactions with these other sets of skills.

### 6.4. Overlap with Executive Processing

Is meta-intelligence the same as executive processing? In the augmented theory of successful intelligence, executive processes are called metacomponents, as described above. They are viewed in the current account as individual constraints, that is, as limiting one’s application of meta-intelligence within the person. They are, however, not the only individual constraints. Others are attitudinal. There are also contextual constraints, which originate outside the person. The bottom line is that meta-components are constraints on the operation of meta-intelligence. They are not meta-intelligence itself. They are a part of meta-intelligence, not the whole thing.

### 6.5. Overlap with Personality

Is meta-intelligence simply another name for personality, or aspects of it? A number of theories have tried, in various ways, to integrate aspects of cognition and personality (e.g., Ackerman 1996; Blömeke et al. 2015; Schneider and McGrew 2018; Sternberg 1997; Ziegler et al. 2012, 2018). Each of these theories attempts the integration in a somewhat different way. At this point in time, it is clear that there is no one consensually accepted framework that specifies exactly how the integration should be achieved. What we believe to be unique in our model are the four particular windows—analytical and practical intelligence, creativity, and wisdom (see also Sternberg 2003)—as a proposed way of looking at their interactions through the use of metacomponents as well as their activation and integration by meta-intelligence.

It is always tempting to reduce new constructs to old ones—in Piaget’s (1972) terms, to assimilate rather than accommodate—but sometimes, it just does not work out well. For example, might the aspect of practical intelligence that resides in one’s ability to persuade others actually be nothing more than the assertiveness facet of extraversion?

The potential problem is that people may be assertive but totally unpersuasive—think of pushy but unpersuasive salespeople who insist you buy their product; or, for some people, the assertive but unpersuasive individual might be their vocal and highly assertive mother- or father-in-law who believes they know how their child’s marriage ought to be and makes sure the married child and son- or daughter-in-law knows it too.

This is not to say that personality is irrelevant to intelligence or meta-intelligence. Openness to experience, for example, has been associated with intelligence in a wide variety of studies (DeYoung 2020). If one defines openness broadly enough, it can account for almost any willingness to do anything one has not done before, but the construct then becomes vacuous and meaningless—not explanatory but rather a catchall category for virtually all motivations to do anything not done before. In the cognition and personality literatures, many different attributes correlate modestly to moderately with each other, a fact recognized long ago by Walter Mischel (1968). Regrettably, such often modest correlations have been too often used to assert causality or even identity (Mackintosh 2011). We have no doubt that selected personality traits would correlate with meta-intelligence, as they correlate with hundreds, if not thousands of other things. They do not reduce to it, however.

## 7. Conclusions

We tend to view constructs such as intelligence, creativity, and wisdom as skills, and in part, almost certainly, they are. However, they are more than skills. Each of them contains an important attitudinal component as well—in particular, the decision to utilize that ability in solving problems of everyday life. This attitudinal and decisional component is more important than ever before, because there are so many contemporary threats to humanity—global climate change, pandemics, bacterial resistance to antibiotics, terrorism, pollution, and a contingent of autocrats and would-autocrats and their sycophantic followers. Schools develop in children attitudes that encourage memory-based and, sometimes, analytical thinking. Such a limited skill and attitude set does not, in itself, suffice in today’s world. The combination of skills and attitudes as applied to problems determines the approach a problem-solver uses.

Skills (or the sets of skills referred to as “abilities’) and attitudes need to go together. Historically, attitudes have been viewed as distinct from skills. However, more and more, researchers are recognizing that skills, in order to be effective, have to be part of a package, a package that includes attitudinal components, as well as personality components (Ackerman and Kanfer 2020). This is especially true of the non-analytical components of this package, which are quite more dependent of personality or attitudinal inclinations. Creativity is in many ways a choice and wisdom rests on value preferences. The laser-like focus of abilities researchers on general intelligence and its factorial elements has led to a narrowing of the lens on the complete set of dispositions needed to make people effective in real-world action as well as to engage in the forms of cooperation that are proper for our species as cultural organisms (Tomasello et al. 2005). Further, skills are often used in ways that result in worse outcomes for the world rather than better ones. This can include when leaders use their skill sets, including cognitive and emotional intelligence, to lie in ways that are effective in convincing followers to listen to them. Through the relation with other people and the broader material and cultural world, the attitude-ability cluster becomes integrated into a wider context. As we have argued throughout this paper, this context constrains and also enables some—but not other—individual problem solving.

To restate, creative, analytical, practical, and wisdom-based approaches are not fully separate from each other. They are integrated through meta-intelligence. In essence, the way in which they differ is in what goal is being pushed or accelerated—creativity, analytical soundness, practicality, achievement of a wise common good—and which forces constrain that goal to ensure that it is achieved in a way that will be successful.

The four constructs of creative, analytical, practical, and wisdom-based approaches are not, strictly speaking, different things. Rather, they represent a common set of processes in which one set of goals is foregrounded and other sets of goals are backgrounded. Our societies, at different times and places, foreground some goals and background other goals, but all four sets of processes work together so that a given idea can withstand the most basic tests of viability. Sometimes, however, what societies foreground or background is not necessarily the most adaptive solution. Indeed, when many societies become totalitarian, they show a disregard the goal of common good. It is interesting that in most of those cases, disregard of wisdom ends up driving those societies to self-destruction (Nazi Germany, Cambodia under the Pol Pot regime, and many others), despite any initial technological or economic boom seen under the first bursts of the totalitarian regime.

The relationships established between creative, analytical, practical, and wisdom-based processes and attitudes are governed by the interactive constraints (emerging at the interface between individual and contextual constraints, as discussed above). These relations can be of *cooperation*, in which the person is compelled, in the situation, to use more than one process or perspective and to integrate their specific demands. This can happen, for instance, on a first date, when people want to present themselves as being open and flexible enough in the way they think and react to a potential partner. However, the relation can also be one of *conflict*, in which situational demands require the person to choose between perspectives; for example, between creativity and analytical intelligence in writing a university essay; between analytical and practical intelligence when asked to quickly solve a problem; or between wisdom and all the other processes when reflecting on a pressing moral dilemma such as those that are presented to health authorities overseeing a global pandemic (or, at a personal level, what to do if one finds a lost wallet).

Such conflicts between perspectives are likely to be experienced intensely by the person as they go against the internal logic of balancing the four windows on problem solving. This balancing is likely to follow cultural norms that favor one course of action and discourage or even condemn others. Fortunately, the most common instances are probably those we call *subordination* in the sense that one (or two, or three) window(es) take(s) the lead, leaving the other(s) in the background. Unlike conflict, subordination is often a matter of preferences and values.

Yet, these preferences to foreground and background different windows for problem solving are not necessarily conscious. Rather, they may be preconscious or even totally unconscious. Sternberg (2018) argued that creative thinking involves three kinds of defiance—defiance of the self, defiance of the crowd, and defiance of the Zeitgeist (or prevailing worldview). One usually is aware of what one thinks and of what others think about an issue. However, the Zeitgeist is a worldview that is so ingrained and socialized into one’s ways of thinking that one is hardly aware of it until it is somehow violated. For example, the COVID-19 pandemic made many people aware of the extent to which basic everyday actions, such as walking around without a mask, shaking hands upon meeting someone, and talking to someone at what previously seemed like an appropriate physical distance, were merely conventions that could be upended in a pandemic. Even here, cultural contexts play a role; preferences for desired personal space is highly culture-specific (Sorokowska et al. 2017). Therefore, adaptive responses to a situation must take into consideration these preferences because they make some choices more likely than others.

Many of our views, similarly, are not in our conscious awareness. For example, democracy around the world once seemed to be on the ascendant. Now it is not so clear (Ziblatt and Levitsky 2018; Mounk 2018). One is continually constrained by assumptions one does not even know one has, whether in thinking about a problem analytically, creatively, practically, or wisely.

Constraints leave their mark on a variety of domains of our existence. Education is one of them. Although one would hope that cooperation between the four perspectives is actively encouraged and supported by teachers, the reality of many classrooms is starkly different. In order to meet economic goals, more and more countries around the world seek to adapt Western models of education as part of their educational reforms, which emphasize the development of memory and analytical skills and attitudes, arguably at the expense of creative, practical, and wisdom-based ones.

Setting aside questions regarding the wisdom and success behind such reforms, Sternberg (2019c) found that teaching for wisdom in basal readers for elementary-school students declined over the course of a century from the beginning of the 20th century to the beginning of the 21st, with an accompanying rise in emphasis on the development of abstract analytical skills and attitudes. Perhaps this change in emphasis mirrored the increase of 30 points in IQ during the 20th century (Flynn 1987). People more and more capitalized on their strengths in analytical skills and attitudes. However, arguably, as the results of enhanced analytical skills and attitudes become more prominent in the world, so did the results of a lack of, and perhaps, declining wisdom (Sternberg 2019a, 2021). The result was that industrial production and many technological developments increased while thoughts about where all these developments were leading us decreased. Life became more comfortable while the environmental conditions that would allow that comfortable life to continue over the long-term degenerated. 

That situation has left us with crises such as that faced by Cheryl that cannot be resolved satisfactorily other than through a combination of creative, analytical, practical, and wisdom-based approaches to problems. Crises are not new; what is new is the sheer power of destruction they can cause. Before worldwide travel, for example, a disease such as COVID-19 probably would have been much more limited in its impact. Terrorism was not exported in the past as it can be today with the development of the Internet.

Meta-intelligence is at least partially teachable. How? By making young people aware that problems can be solved through multiple windows and their interactions. Such teaching may not result in extraordinary increases in meta-intelligence. However, our schooling over-emphasizes the window of analytical intelligence. Simply teaching students that they have creative, practical, and wisdom-based options, and teaching them what these options are (Sternberg et al. 2009) may encourage them to approach problems in ways that they otherwise would not have thought to utilize.

In the case of Cheryl and Vaccimax, her attitudes will have a major effect on how she responds to Vaccimax’s problems. What kinds of considerations does she view as important? What kinds are unimportant? What kinds need to be attended to right away, and what kinds can be deferred? What would the cultural context and the situation she is in at that time push her towards or away from?

Leaders have not always felt the need to tell the truth. However, today, more than ever, their shameless lying is being amplified by cynical manipulators of social media and other Internet-based means of communication. Threatening the Enlightenment values on which modern societies are built, manipulators make a concerted effort to blur the distinction between truth and falsehood, sense and nonsense.

Rising IQs apparently have been essentially useless in protecting people against the onslaught of misinformation and, in many cases, the willingness to believe it. In addition, suppressing educational access to critical thinking has been always a strategy of authoritarian regimes. It is not enough to have the analytical ability to discern that information is false or misleading; the creative ability to avoid blind conformity; the common sense to recognize when someone is lying right to your face; and the wisdom to appreciate that cynical leaders care only about themselves, not their followers. Some leaders today (and in the past) have encouraged followers to look through none of the windows for problem solving, but rather, simply to obey the leader’s dictates. They lead by slogans, often ones that appeal to people’s sense of victimization. And they appeal to raw emotions rather than to reason.

People need to make the decision to actively coordinate and deploy their creative, analytical, practical, and wisdom-based skills and attitudes through meta-intelligence--to stand against the forces that are destructive of intellectual and creative freedom. Often, it is easier just to go along with mass movements—we saw this during the rise of fascism during World War II—and we are seeing it again today as people are blind or even welcoming of authoritarian leaders, whether in politics, business, or even science, who want not so much to destroy people’s intellectual skills and attitudes as to anesthetize them—rendering them inoperable and inert so that they are not deployed. This is also true for long-term environmental changes caused by widespread denial of the consequences in nature of unchecked industrialization and mass consumption, and a worsening economic- equality system that enables a tiny number of people to control an enormous range of the world’s resources. It is clearer every day that economic growth, to be sustainable, requires an understanding of what skills are foregrounded and what skills are backgrounded. Further, what looks bright on the one side may have another darker side that, if left unattended, can erode any other gain we have made in human societal development.

The bottom line is that problem solving needs to be viewed not only as a set of skills but also as a meta-intellectual attitude to effectively deploy those skills. Attitudes, in turn, are informed by choices. Additionally, choices are shaped by values and a wider sociocultural context. If we do not address what we value and why, we will not understand what we choose and why, and how these choices will determine our skills and what skills we foreground and what ones we background. Today, the stakes are high. We risk ending up with stereotypical members of high-IQ societies whose main accomplishment in life is to join a high-IQ society whilst our environment collapses and our democratic institutions degrade. Intelligence should be viewed, as Binet and Simon (1916) recognized, as comprising in part the initiative to deploy skills in problem solving so as broadly to adapt to the environment in useful and effective ways.

This systemic embedding of contexts makes the issue of assessment all the more interesting and invites researchers to move past traditional personality and abilities tests alone and toward mixed-method designs in which person and context can be studied both quantitatively and qualitatively, especially in the case of naturalistic decision making. Researching the articulation between individual-level variables and sociocultural variables requires also longitudinal designs offering the possibility of studying the person in various context and at multiple moments in time.

Returning to Cheryl, we can ask whether Cheryl will effectively solve her problems at Vaccimax. The answer depends on context and on her skills, for sure—as well as the advice from those around her. However, it also depends on her attitudes, and especially, her willingness to deploy her skills and attitudes effectively to look through multiple windows to see how to approach—in particular, how to recognize, define, and solve the problems of Vaccimax. We all need creative, analytical, practical, and wisdom-based skills and attitudes to open the windows that will enable us to look upon problems and solve them in a manner that is sensitive not only to our own needs, but to the needs of others as well.

## Figures and Tables

**Table 1:** Metacomponents Applied to Analytical, Creative, and Practical Intelligence, as well as Wisdom.

Aspect of Intelligence/Metacomponent	Analytical Intelligence	Creative Intelligence	Practical Intelligence	Wisdom
**Recognizing the Existence of a Problem**	I am miserable in my job and can’t think in this environment	I am miserable in my job and I can’t be creative in this environment	The environment in which I am working is interpersonally toxic	I cannot do the company or anyone any good if I am in a toxic environment
**Definition of the Problem**	The environment is toxic and my salary is inadequate to meet my family’s financial needs	I can make the environment less toxic by distancing myself from toxic coworkers or I can transfer; I can either ask for a promotion or a raise	When I am in a toxic environment, my work suffers and so does my health and sense of psychological wellbeing	Over the long term, my ability to do good for the company or the world will suffer if I cannot find a better environment in which to work
**Allocating Resources to the Solution of the Problem**	I need to do whatever it takes to make things better because I can’t go on this way	I need to do whatever it takes to make things better because I can’t go on this way	I need to spend a substantial amount of time I don’t think I have to deal with this problem	I need to devote resources to find an environment where I can help people
**Mentally Representing the Problem**	I need either to make the environment less toxic and my pay higher or I need to find a new job	I can move to a different office down the hall or to the finance division or I can put my resume on job sites	Outline the options—stay in the job and suck it up; or shape the environment to be less toxic; or find another environment in which to work	I am constantly under attack and I am spending time fighting off attacks rather than doing constructive world-changing work
**Formulating a Strategy for Problem Solution**	I can ask to transfer to another division; I can ask for a raise; I can ask for a promotion	I can explain to my boss that I need either to change my coworker and boss or find a new job	First seek to find the cause of the toxicity and see whether it can be reduced; if not, seek another job	I need to find a solution that will not only help me personally but enable me to fulfill my destiny to help others
**Monitoring Problem Solving While It is Ongoing**	Once they are implemented, I can see whether any of these strategies are working	Are random-assignment, double-blinded vaccine trials succeeding in Phases 1, 2, 3?	Is the environment becoming less toxic, and/or am I getting job interviews?	Am I helping the organization and the world any more at this point?
**Evaluating Problem Solution**	I will see whether I made the environment less toxic and got a raise or whether I was able to find a new job	Evaluate treatment efficacy and cost after Phase 3 is completed	Did I succeed in making the environment less toxic or in finding another job?	Have I become a fully functioning and wise worker doing what I came here to do—change the world for the better?
